# Supplementary material for: Increased formate overflow is a hallmark of oxidative cancer
Source: Nat Commun. 2018 Apr 10;9:1368. doi: 10.1038/s41467-018-03777-w (PMC5893600; doi:10.1038/s41467-018-03777-w)
Supplement: Supplementary file 1 — Supplementary Information [file 41467_2018_3777_MOESM1_ESM.pdf]

Increased formate overflow is a hallmark of oxidative cancer

Supplementary Information

Meiser et al.

List of Supplementary Information

Supplementary Figures 1 to 11

Supplementary Table 1

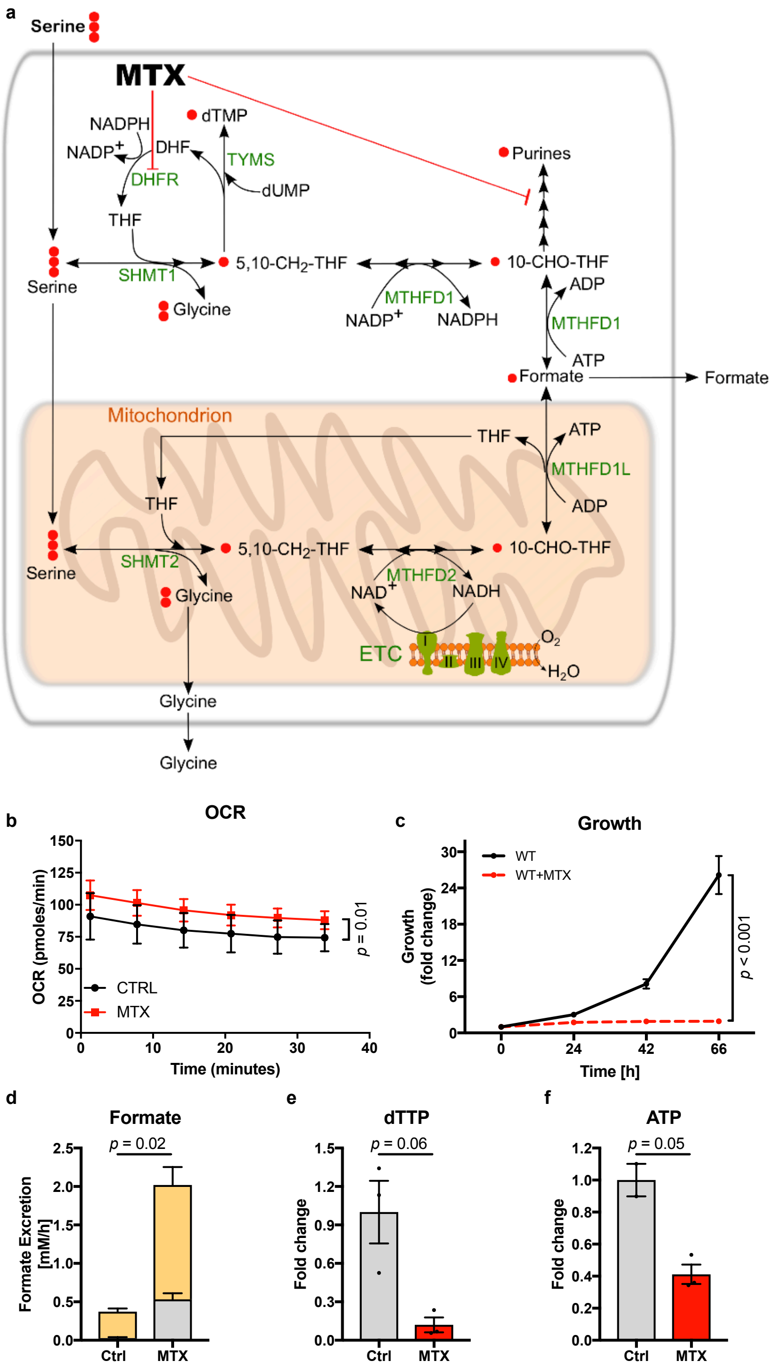

Supplementary Figure 1: Methotrexate does not inhibit serine catabolism. (a)Model illustrating a possible mechanism that allows serine catabolism in the mitochondria during nucleotide synthesis inhibition through methotrexate (MTX) in the cytoplasm. (b) Oxygen Consumption rate (OCR) upon Methotrexate (MTX) treatment (50nM). Cells have been treated with or without MTX at the time of OCR measurement. Results are obtained from one experiment with 10 replicate wells. Error bars denote standard deviation of technical replicates. (c) Growth assay with HAP1 cells with MTX (50nM) compared to untreated cells. (d-f) Effects of MTX on cellular metabolism assessed by: (d) formate release rates of cells cultured in [U-^{13}C]serine and (e,f) intracellular dTTP (e) and ATP (F) levels. Except of b, results were obtained from three independent experiments (n=3) with triplicate wells each. Error bars denote s.e.m. *p*-value is calculated using an unpaired *t*-test with Welch's correction (in d-f) and by multiple *t*-test in b and c.

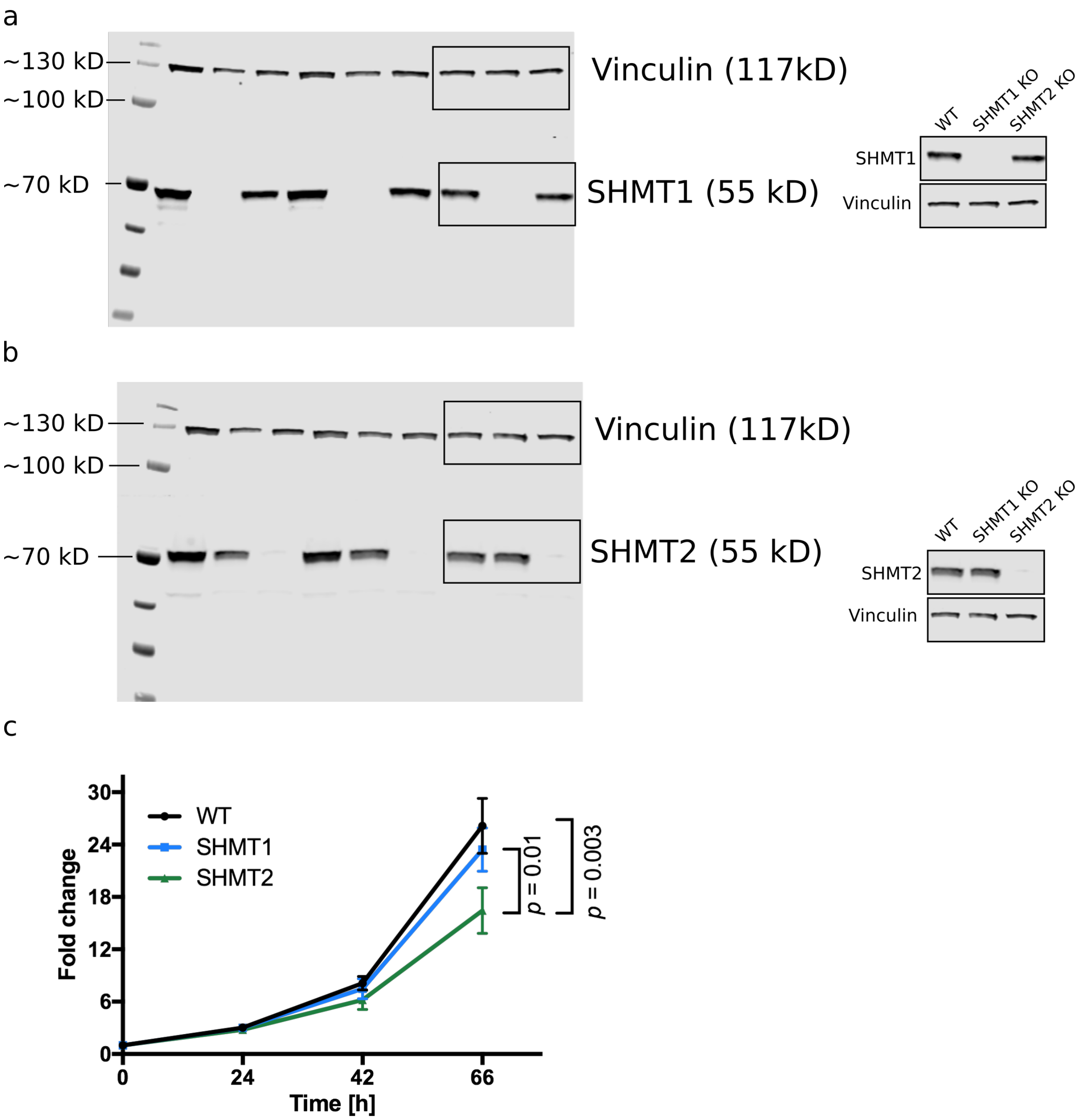

Supplementary Figure 2: Western Blot (a, b) to confirm *SHMT1* and *SHMT2* knockout in HAP1 cells used in Figure 1 and (c) growth assay for HAP1 WT, SHMT1 and SHMT2 KO cells. Results were obtained from three independent experiments (n=3). Error bars indicate s.e.m. *p*-value is calculated using a multiple *t*-test.

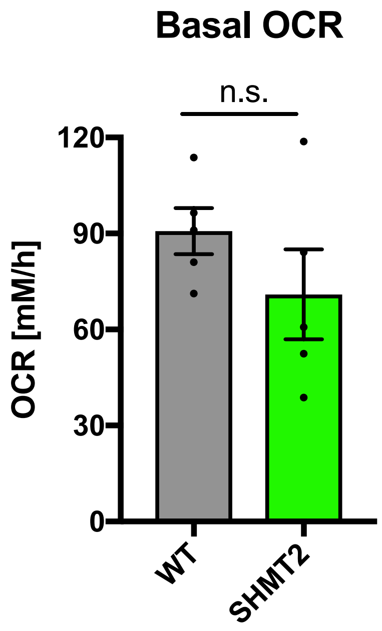

Supplementary Figure 3: Oxygen consumption rate of HAP1 WT and SHMT2 KO cells. Each dot indicates one independent experiment (n=5). Error bars indicate s.e.m.. There is no statistical significant difference (using an unpaired *t*-test with Welch's correction).

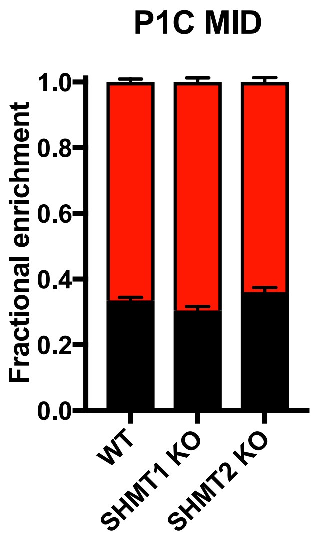

Supplementary Figure 4: Comparison P1C in WT, SHMT1 KO and SHMT2 KO. Results were obtained from 11 independent experiments with triplicate wells each (n = 11). Error bars indicate s.e.m.

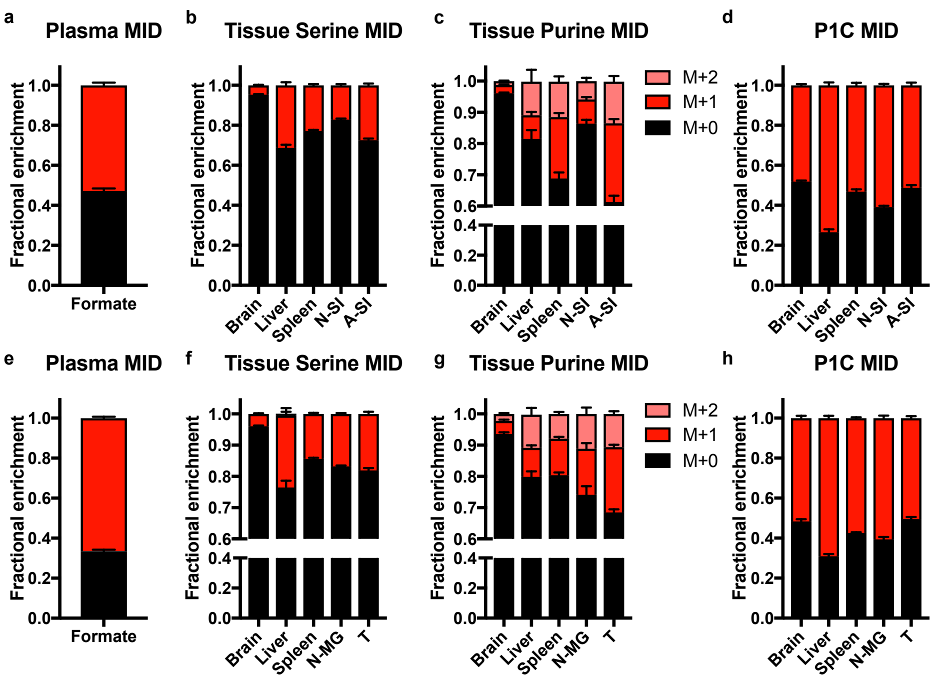

Supplementary Figure 5: *In vivo* labeling pattern in *APC*$^{Min/+}$ and PyMT mice 24 h after ^{13}C MeOH injection. (a-d) Labeling pattern in APC$^{Min/+}$ mice at clinical endpoint (a) MID of plasma formate, (b) Serine MID, (c) Purine MIDs and (d) P1C in purines in different tissues (as indicated). (e-h) Labeling pattern in PyMT mice at clinical endpoint (as in a-d). Results were obtained from at least three mice. Error bars denote s.e.m.

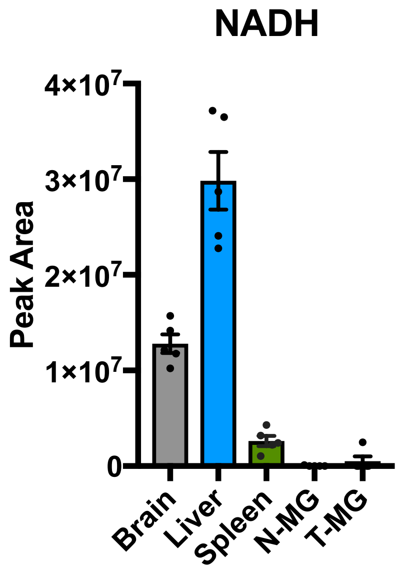

Supplementary Figure 6: NADH peak areas in different tissues (as indicated) of PyMT end-stage mice. Results were obtained from five mice. Error bars denote s.e.m. N: normal; T: tumor; MG: mammary gland.

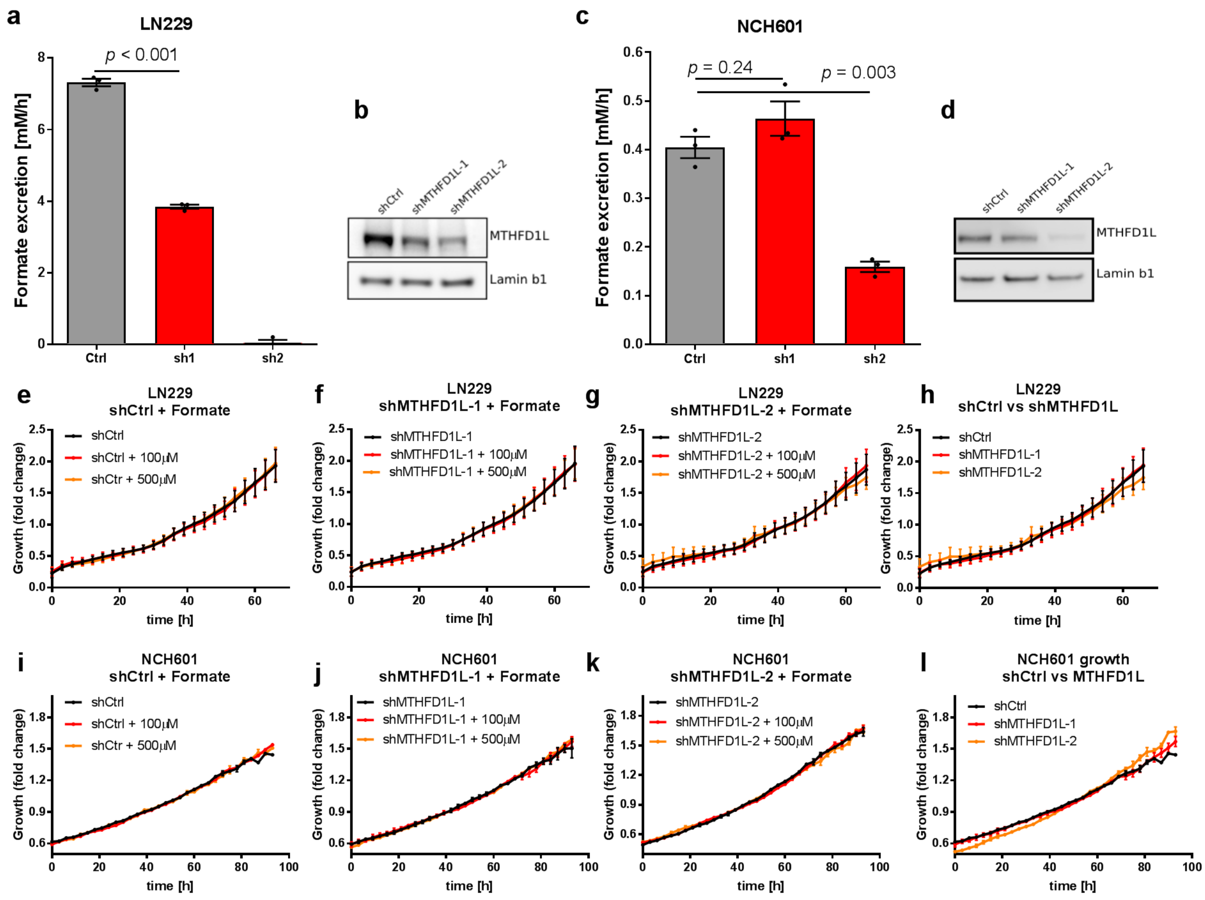

Supplementary Figure 7: Knockdown validation in LN229 and NCH601 glioma cell lines. (a-b). LN229 (a) medium release rates of formate in shCtrl, shMTHFD1L-1 and -2 cells and (b) a representative Western Blot to confirm knockdown at protein level. (c-d) Respective analysis as in a and b for NCH601 cells. (e-l) Comparison of growth rates in LN229 (e-h) and NCH601 (i-l) for the three different shRNA cell lines to each other (h and l) and in respect to different formate concentrations (e-g and i-k). Results were obtained from three independent experiments (n=3) with triplicate wells each in a and c and four replicate wells each in e-l. Error bars denote s.e.m. *p*-value is calculated using an unpaired *t*-test with Welch's correction.

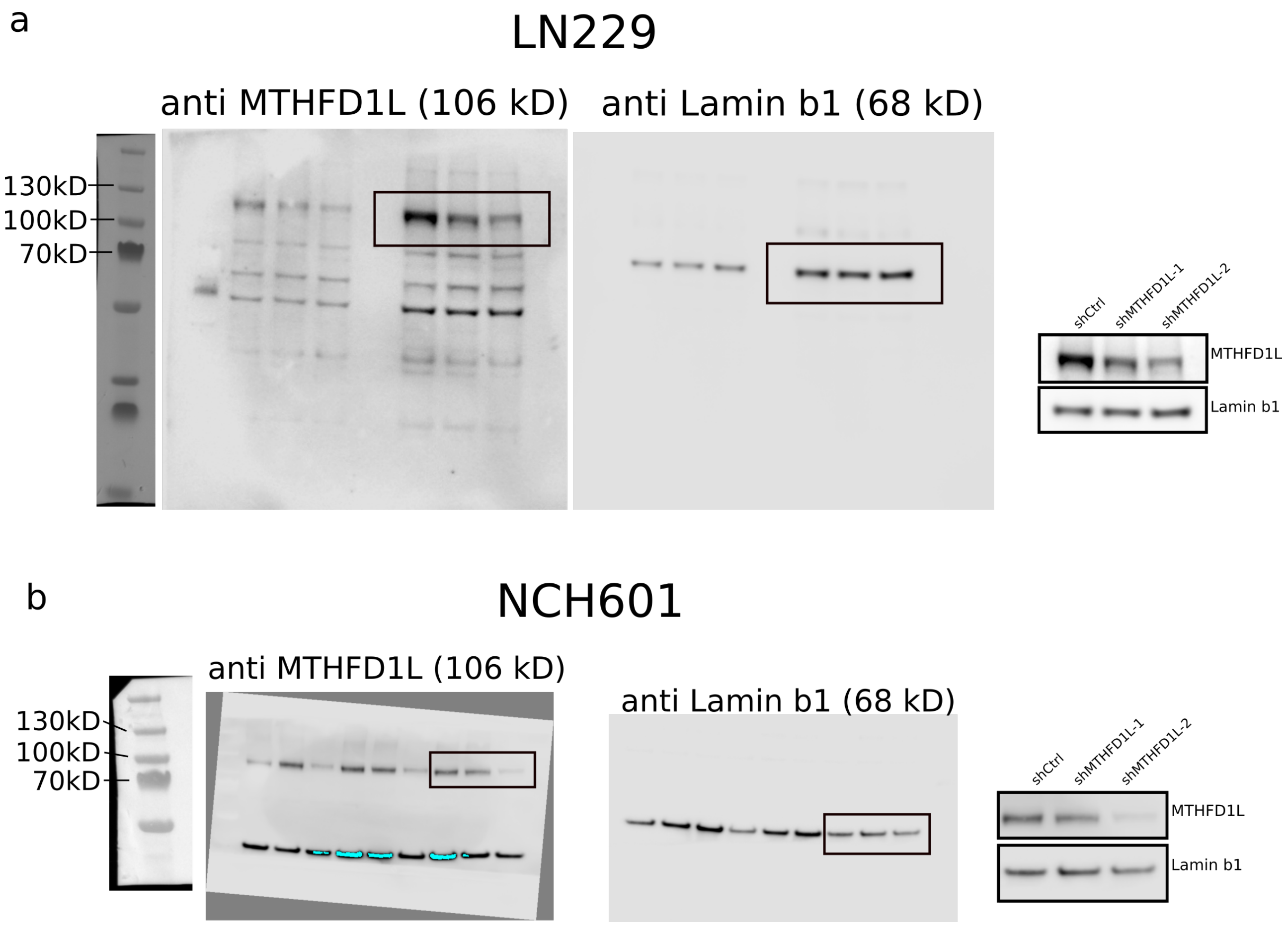

Supplementary Figure 8: Knockdown validation in LN229 and NCH601 glioma cell lines. Uncropped Western Blot images. a: LN229; b: NCH601

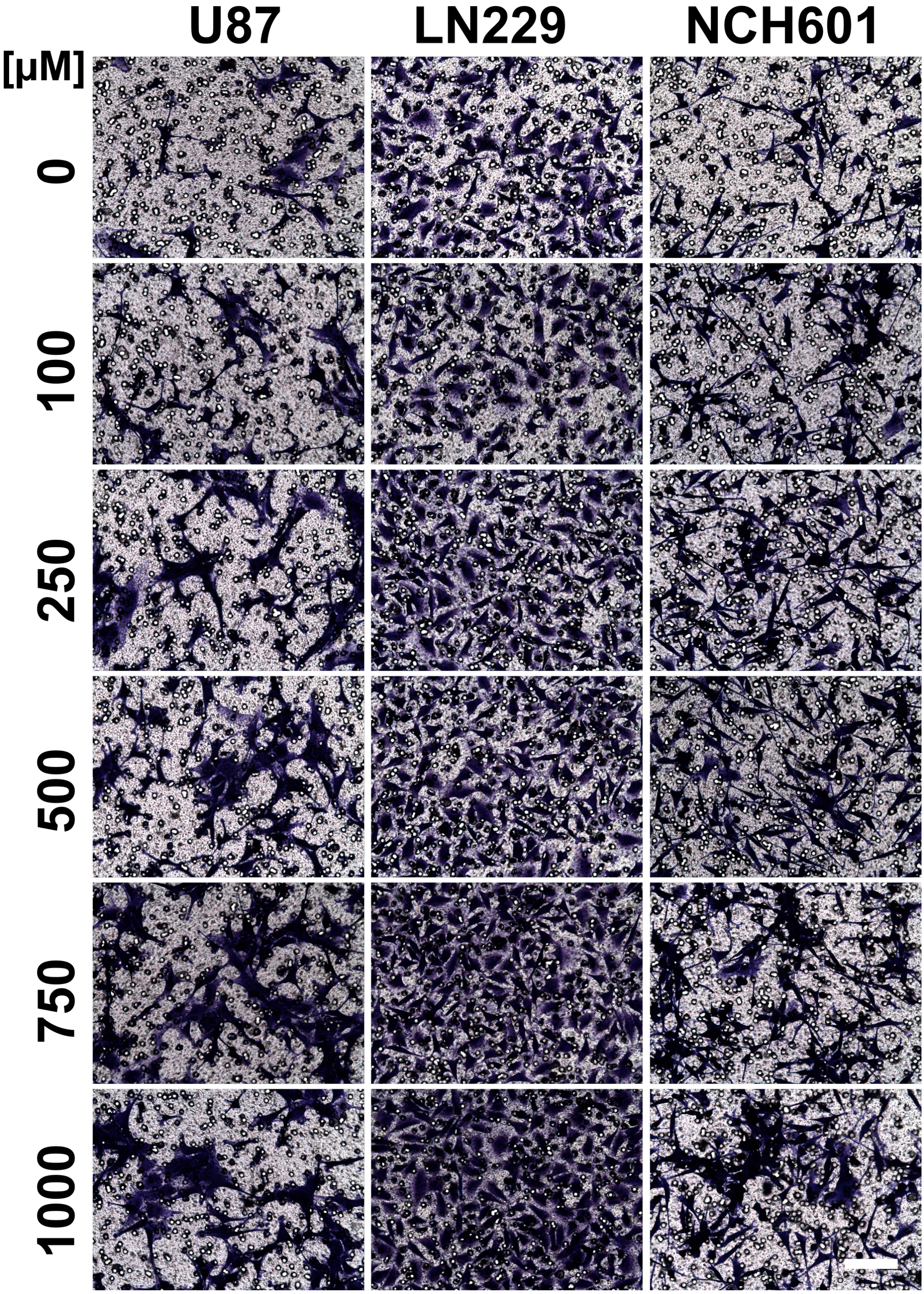

Supplementary Figure 9: Representative microscopy images of Boyden chambers with U87, LN229 and NCH601 at different Na-formate concentrations. Cells were stained with crystal violet. Scale bar: 100 µm

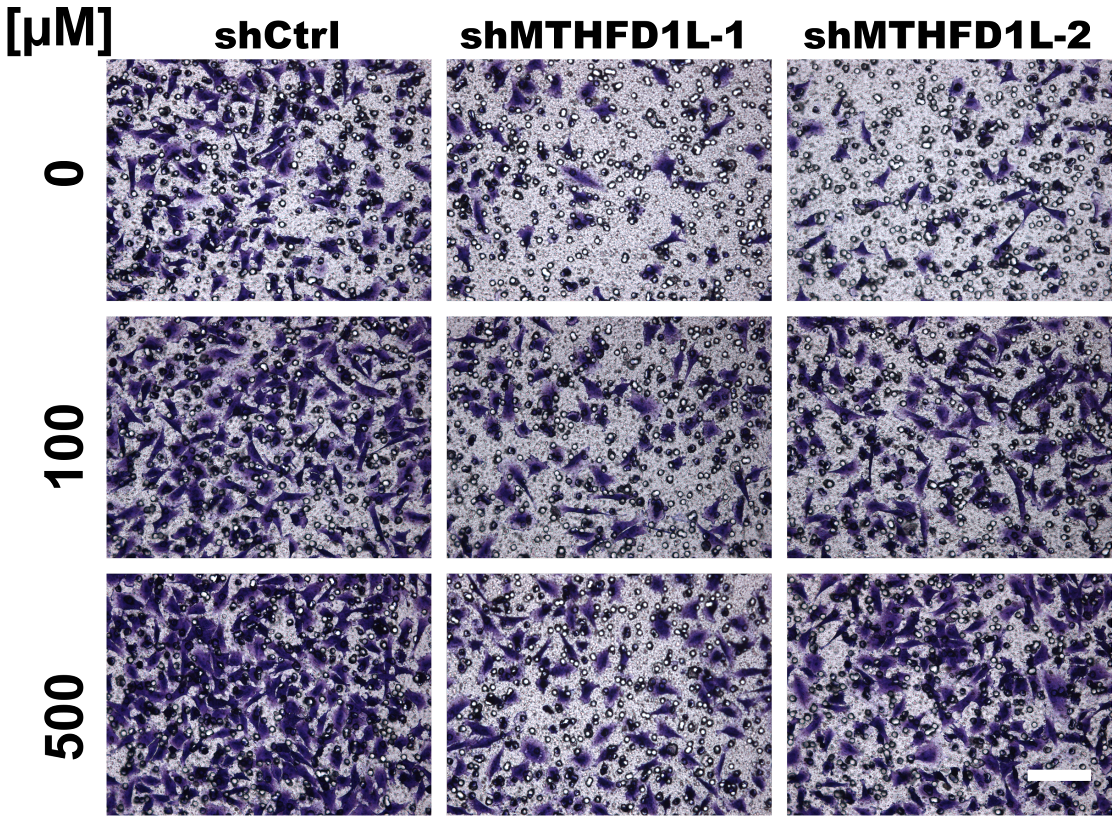

Supplementary Figure 10: Representative microscopy images of Boyden chambers with LN229 cells. Cells were stained with crystal violet. Scale bar: 100 µm.

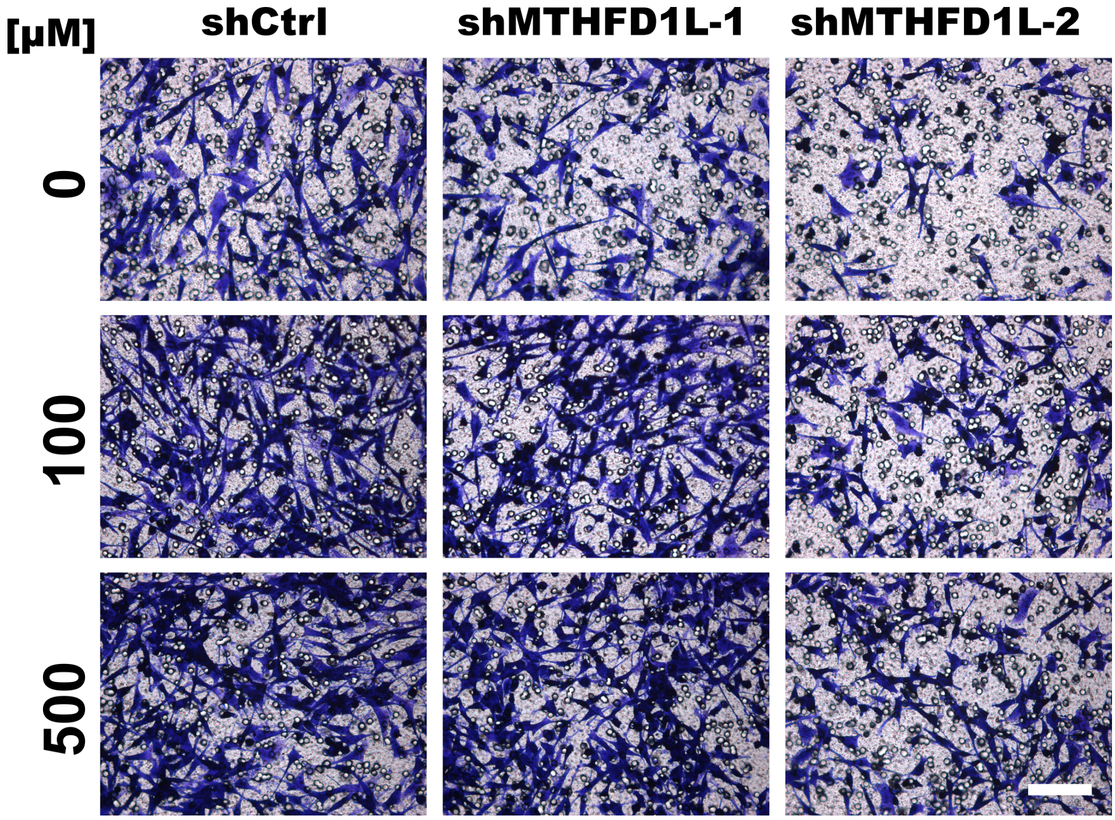

Supplementary Figure 11: Representative microscopy images of Boyden chambers with NCH601 cells. Cells were stained with crystal violet. Scale bar: 100 µm.

Symbol	Cell line	Cancer type	Number of experiments
Circle, black	A549	Lung	2
Circle, red	A549+Rotenone	Lung	2
Square, black	G-CCM	Grade 3 glioma	1
Diamond, black	HAP-1	Myeloid leukemia	6
Triangle-down, black	HCT116	Colorectal	2
Triangle-down, red	HCT116+Rotenone	Colorectal	2
Hexagon, black	LN229	Glioblastoma	3
Circle black with grey outline	MDA-MB468	Breast	1
Triangle-up, black	U87	Glioblastoma	2

Supplementary Table 1: Cell line panel analyzed and used for correlation analysis in figure 1b.
